# Full-endoscopic trans-pars interarticularis discectomy for foraminal and extraforaminal lumbar disc herniation: surgical technique and early clinical outcomes

**DOI:** 10.1016/j.bas.2026.106058

**Published:** 2026-04-17

**Authors:** Mohammad Badra, Georges Sakhat, Ahmad Haj Hussein, Ralph Maroun, Ramzi Moucharafieh, Karim Areslan, Youssef Jamaleddine

**Affiliations:** a Orthopedic Surgery and Traumatology, University of Balamand, Tripoli, Lebanon; b Clemenceau Medical Center, Beirut, Lebanon; c Lebanese American University Medical Center-Rizk Hospital, Beirut, Lebanon; d Department of Orthopaedic Surgery, Lebanese University, Beirut, Lebanon; e Department of Orthopedic Surgery, Lebanese American University Medical Center-Rizk Hospital, Beirut, Lebanon

**Keywords:** Lumbar vertebrae, Endoscopy, Discectomy, Minimally invasive surgery, Trans-pars approach

## Abstract

**Introduction:**

Foraminal and extraforaminal lumbar disc herniations compress the exiting nerve root, causing radiculopathy. Open approaches may require facetectomy with risk of instability, while transforaminal endoscopy places instruments near the dorsal root ganglion, potentially causing postoperative dysesthesia.

**Research question:**

Is full-endoscopic trans-pars interarticularis discectomy a safe and effective treatment for foraminal and extraforaminal lumbar disc herniations?

**Materials and methods:**

This retrospective case series analyzed prospectively collected data from patients who underwent full-endoscopic trans-pars interarticularis discectomy at a single center for foraminal and extraforaminal disc herniations. Seventeen patients with complete preoperative and 6-month data were included. Outcomes were evaluated using VAS scores for leg and back pain, the Oswestry Disability Index (ODI), and the Short-Form 12 (SF-12) questionnaire. Complications and satisfaction were documented. Pre- and postoperative outcomes were compared statistically.

**Results:**

No intraoperative complications occurred. Postoperatively, there were no infections, bleeding, new neurological deficits, or recurrent herniations. Two patients (11.8%) developed transient leg dysesthesia. At 6 months, VAS leg pain improved from 8.47 ± 1.91 to 2.24 ± 2.02 and VAS back pain from 7.82 ± 2.30 to 2.06 ± 1.25 (both p < 0.001). ODI decreased from 53.53 ± 19.06 to 29.18 ± 16.25 (p < 0.001) and SF-12 physical and mental component scores improved significantly.

**Discussion and conclusion:**

The full-endoscopic trans-pars interarticularis approach appears to be a safe and effective alternative for foraminal and extraforaminal disc herniations, achieving meaningful improvements in pain, disability, and quality of life with low morbidity. Larger prospective studies with longer follow-up are needed to confirm these findings and refine indications.

## Highlights

• Open surgery risks instability; transforaminal endoscopy risks dysesthesia.
• Trans-Pars endoscopy offers a safe corridor and early nerve root visualization.
• In 17 cases: no intraoperative or major postoperative complications observed.
• Leg dysesthesia occurred in 2 patients (11.8%) and resolved conservatively.
• Significant 6-month pain and function improvements (VAS, ODI, SF-12; p < 0.001).

## 1 Introduction

Foraminal and extraforaminal lumbar disc herniations (LDH) are a distinct subset of lumbar disc herniation that compress the exiting nerve root and often the dorsal root ganglion, producing severe radiculopathy and sensory disturbances (Epstein, 2002). Contemporary and earlier studies place the proportion of far-lateral LDH proportion at roughly 7-12% of all lumbar disc herniations (Abdullah et al., 1974; Kerr et al., 2024).

Surgical management of extraforaminal disc herniations poses significant technical challenges. Traditional open approaches can achieve adequate decompression of the affected nerve root but often necessitate partial or complete facetectomy, potentially compromising segmental stability and predisposing to iatrogenic spondylolisthesis. These limitations have driven the evolution of less invasive, tissue-preserving techniques (Zeng et al., 2017).

The full-endoscopic transforaminal discectomy, performed through Kambin's triangle, provides a percutaneous and muscle-sparing alternative. However, because the working sheath passes in close proximity to the exiting nerve root and dorsal root ganglion, patients may experience postoperative dysesthesia (Fanous et al., 2019; Lewandrowski et al., 2020a). The procedure becomes particularly challenging at the L5-S1 level, where anatomical constraints such as a high iliac crest, prominent sacral ala, broad facets, and a narrowed foramen can restrict the standard transforaminal trajectory, necessitating modified surgical approach (Reulen et al., 1996; Mirkovic et al., 1995; Ebraheim et al., 1997). To address these challenges, the trans-pars interarticularis endoscopic approach has been introduced. This technique utilizes consistent bony landmarks at the pars interarticularis, allows early identification of the exiting nerve root, and minimizes bone removal, thereby reducing neural manipulation and potential injury (Greil et al., 2023).

In this study, we present the clinical outcomes of patients who underwent a stepwise, full-endoscopic trans-pars interarticularis extraforaminal discectomy, and we provide a detailed description of the surgical technique.

## 2 Materials and Methods

This study was conducted in compliance with the principles of the Declaration of Helsinki. The study's protocol was reviewed and approved by the Institutional Review Board of Clemenceau Medical Center, Beirut (Ref: ERRC/SU/12/2025). Written informed consents were obtained from all patients for inclusion in the study.

This is a single-center retrospective study on prospectively collected data. The authors reviewed adult patients with foraminal or extraforaminal lumbar disc herniation who underwent full-endoscopic trans-pars interarticularis discectomy at our institution and for whom preoperative and 6-month postoperative questionnaires were available. Seventeen patients met these criteria and were included in the analysis. Sociodemographic and clinical variables (age, sex, body mass index, smoking and alcohol status, work type, comorbidities, symptom duration, presence of neurological motor deficit, herniated disc level and location, and prior steroid injections) were obtained from the prospective database and the patients’ medical records. Pain intensity was evaluated using 0-10 visual analogue scales (VAS) for leg and low back pain. Disability was assessed with the Oswestry Disability Index (ODI) (Fairbank et al., 1980), and health-related quality of life with the 12-Item Short Form survey (SF-12) (Ware et al., 1996), yielding physical (PCS) and mental (MCS) component scores. At 6 months post-operatively, patients also rated their satisfaction with surgery and whether they would recommend or repeat endoscopic discectomy. All questionnaires were completed preoperatively and at 6 months postoperatively as part of routine follow-up.

The normality of the data was assessed using the Shapiro test. Paired sample *t*-test and Wilcoxon signed-rank test were used as appropriate to compare the pre-operative and post-operative means of the dependent variable. P-value <0.05 was considered statistically significant.

All statistical analyses were performed using IBM SPSS Statistics for Windows, Version 27.0. Armonk, NY: IBM Corp.

## 3 Surgical technique

**Fig. 1:**
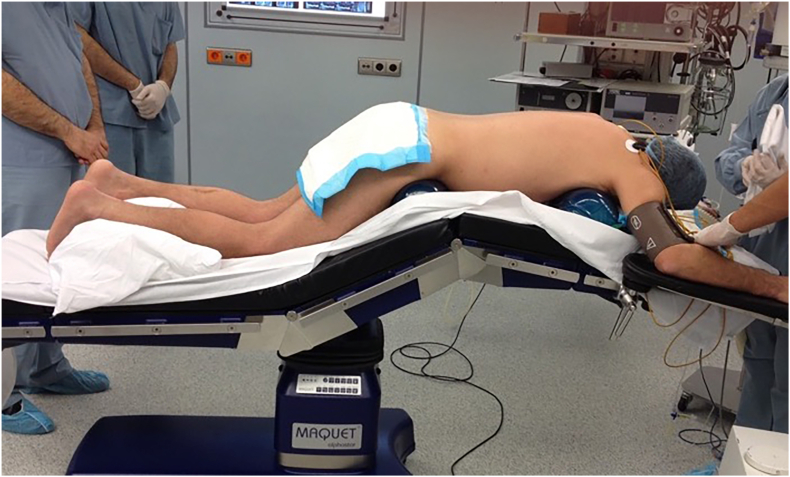
Patient placed in prone position with hips and knees flexed on a radiolucent table to decrease lumbar lordosis and widen the intertransverse space for the full-endoscopic trans-pars interarticularis extraforaminal approach.

All procedures were performed with the patient in the prone position on an adjustable radiolucent operating table under general anesthesia. The table was flexed at the hips and knees to decrease lumbar lordosis and widen the intertransverse process space (Fig. 1).

**Fig. 2:**
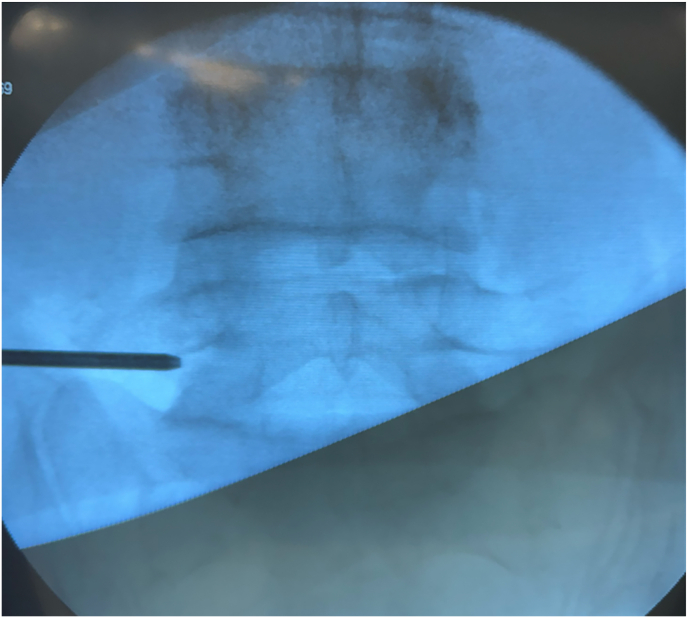
Intra-operative fluoroscopic anteroposterior view showing the skin entry point at the intersection of the lateral border of the pars interarticularis and the caudal margin of the corresponding pedicle.

Under fluoroscopic guidance, the skin entry point was identified at the intersection of the lateral border of the pars interarticularis and the caudal margin of the corresponding pedicle (Fig. 2).

**Fig. 3:**
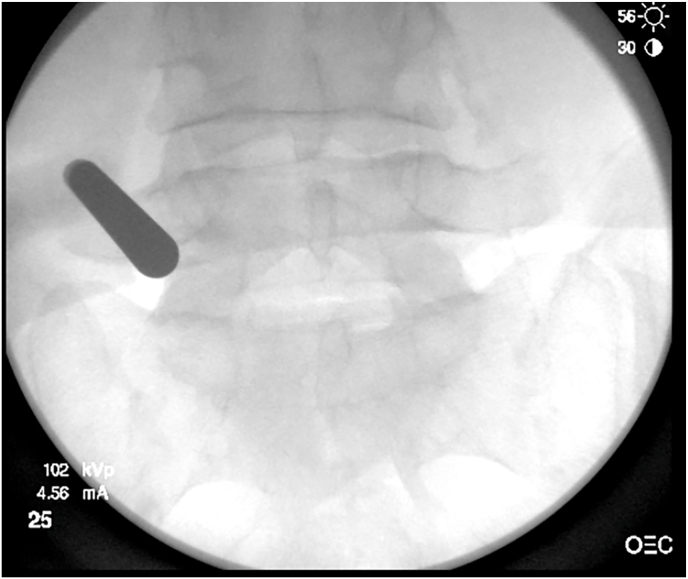
Intra-operative fluoroscopic anteroposterior view showing docking of the dilator and working cannula on the lateral pars-transverse process junction with endoscopic soft-tissue clearance using the RIWO spine system.

Using the Riwo Spine system (Knittlingen, Germany), a dilator was introduced and docked on the lateral surface of the pars interarticularis to bluntly detach and clear the overlying soft tissues (Fig. 3).

The working cannula was then advanced over the dilator, which was subsequently removed, allowing insertion of the endoscope.

**Fig. 4:**
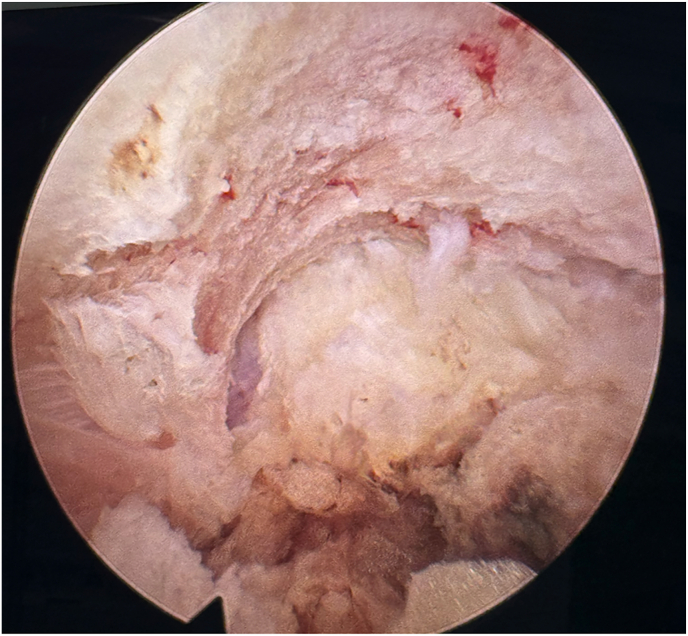
Endoscopic view after limited pars and transverse process resection demonstrating the osseous working corridor and exposure of the superior margin of the extraforaminal ligamentum flavum.

Soft tissue dissection was completed using a radiofrequency bipolar probe to expose the lateral margin of the pars and the inferomedial border of the superior transverse process. The bony junction between the superolateral aspect of the pars and the inferomedial aspect of the transverse process was then identified. Using endoscopic burrs and Kerrison rongeurs, limited bone resection was performed at this corner to visualize the superior margin of the extraforaminal ligamentum flavum (Fig. 4). In certain cases, the tip of the superior articular process of the inferior vertebra was partially resected to improve visualization of this area.

**Fig. 5:**
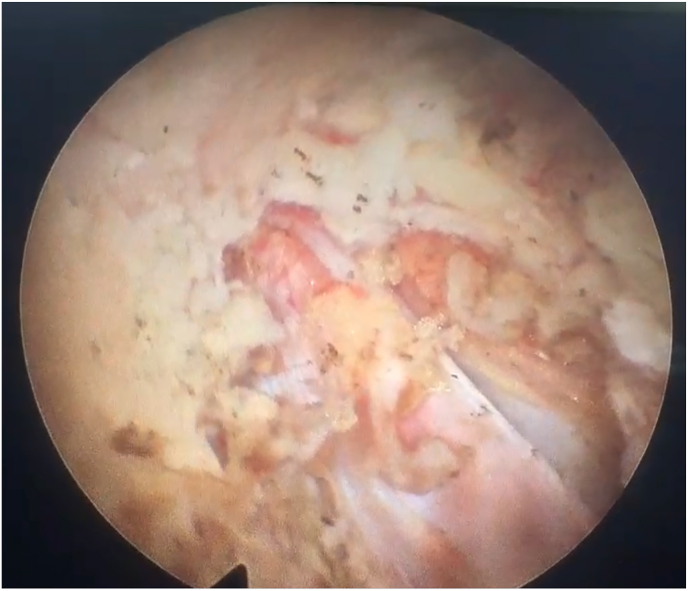
Endoscopic view after targeted flavectomy showing the extraforaminal segment of the exiting nerve root surrounded by perineural fat.

Resection of the targeted portion of the ligamentum flavum exposed the perineural fat surrounding the extraforaminal segment of the exiting nerve root. Gentle lateral dissection was carried out to fully delineate the nerve root (Fig. 5). The herniated disc fragment was then identified and removed. Using an endoscopic hook or probe, the nerve root was gently mobilized to inspect for residual disc material, including at its cranial aspect. Upon completion, the endoscope and working cannula were withdrawn, and the skin was closed with a single suture.

**Fig. 6:**
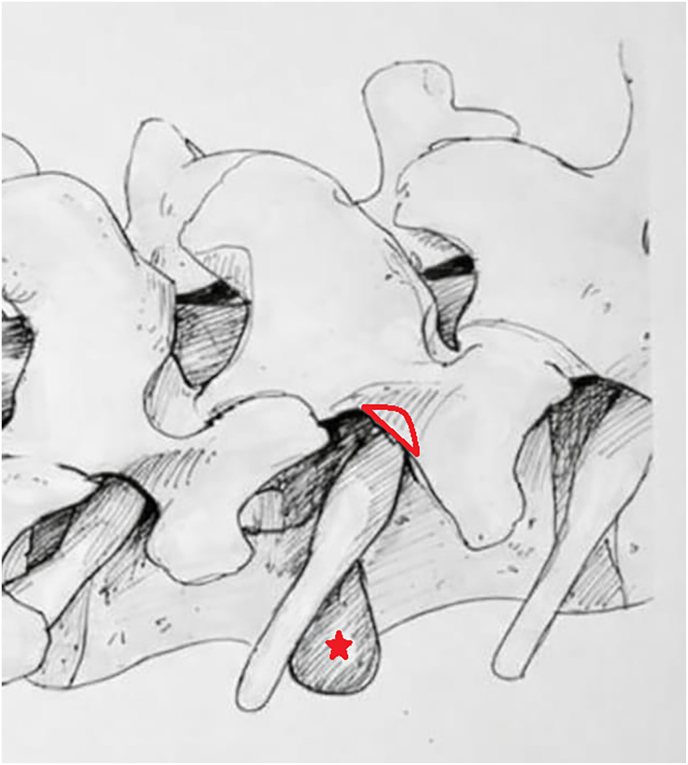
Schematic of the full-endoscopic trans-pars interarticularis corridor to the foraminal or extraforaminal zone. *A small, focal cortical window (red wedge) is created at the pars-transverse process junction to reach the foraminal or extraforaminal space and remove the disc fragment (red star), while preserving the pars and facet joint.*

To facilitate three-dimensional understanding of the trans-pars approach and the anatomic relationships at the pars-transverse process junction, a schematic illustration of the working corridor and the site of minimal corner-only cortical resection is provided (Fig. 6).

## 4 Results

Seventeen patients were included, with patient characteristics summarized in Table 1.

**Table 1:** Sociodemographic and medical characteristics of included patients.

Characteristics	n (%) or mean ± standard deviation
Age (years)	60.88 ± 13.62
Gender	
Male	14 (82.4%)
Female	3 (17.6%)
Body Mass Index (kg/m^2^)	31.65 ± 10.7
Work Type	
I am a desk worker	4 (23.5%)
I work but I'm not a desk worker	9 (52.9%)
I don't work	4 (23.5%)
Smoker	
No	8 (47.1%)
Yes	9 (52.9%)
Alcohol	
No	12 (70.6%)
Yes	5 (29.4%)
Comorbidities^a^	
None	3 (17.6%)
Yes	14 (82.4%)
Symptom Duration Pre-operatively	
Less Than 1 Month	3 (17.6%)
1-6 Months	9 (52.9%)
6-12 Months	3 (17.6%)
1-2 Years	1 (5.9%)
>2 Years	1 (5.9%)
Neurological (motor) deficit	
No	9 (52.9%)
Yes	8 (47.1%)
Herniated disc level	
L2-L3	1 (5.9%)
L3-L4	3 (17.6%)
L4-L5	10 (58.8%)
L5-S1	3 (17.6%)
Herniated disc location	
Foraminal	8 (47.1%)
Extraforaminal	9 (52.9%)
Previous steroids injection	
None	7 (41.2%)
One Time	6 (35.3%)
Two Times	3 (17.6%)
More Than Three Times	1 (5.9%)

a Comorbidities include hypertension, diabetes mellitus, dyslipidemia, cardiovascular disease, cancer, chronic lung disease, irritable bowel disease, renal Failure, hypothyroidism, or hyperthyroidism.

Symptoms were present for 1-6 months in nine patients (52.9%), while three (17.6%) had symptoms for less than 1 month, three (17.6%) for 6-12 months, and two (11.8%) for at least 1 year. A pre-operative motor deficit was present in eight patients (47.1%). The index level was L4-L5 in ten patients (58.8%), L3-L4 and L5-S1 in three patients each (17.6%), and L2-L3 in one patient (5.9%). Herniation was foraminal in eight cases (47.1%) and extraforaminal in nine (52.9%). Ten patients (58.8%) had received at least one epidural steroid injection before surgery. These findings are summarized in Table 1.

No intraoperative complications occurred, with no dural tears or nerve injuries. Postoperatively, there were no cases of wound infection, bleeding, new paralysis, recurrent herniation at the index level, or worsening of back pain or motor weakness. Leg dysesthesia in the distribution of the operated nerve root was reported by 2 patients (11.8%). At 6 months, 11 patients (64.7%) were very satisfied and 5 (29.4%) were satisfied with the surgical outcome, while 1 (5.9%) was neither satisfied nor dissatisfied; none were dissatisfied. Sixteen patients (94.1%) stated they would recommend endoscopic discectomy to someone with similar symptoms, and 13 (76.5%) would undergo the same procedure again if needed. These findings are summarized in Table 2.

**Table 2:** Surgical characteristics of the included patients.

Characteristics	*n* (%)
Intraoperative Complications	
Dural tear	0 (0.0%)
Nerve injury	0 (0.0%)
Postoperative Complications	
Bacterial infection	0 (0.0%)
Bleeding	0 (0.0%)
Paralysis	0 (0.0%)
Recurrent herniation (same level)	0 (0.0%)
Increase in back pain	0 (0.0%)
Increase in muscle weakness	0 (0.0%)
Leg dysesthesia	2 (11.8%)
Satisfaction level	
Very satisfied	11 (64.7%)
Satisfied	5 (29.4%)
Neither satisfied nor dissatisfied	1 (5.9%)
Would You Recommend Endoscopic Discectomy?	
Yes	16 (94.1%)
Maybe	1 (5.9%)
No	0 (0.0%)
Would You Repeat Endoscopic Discectomy?	
Yes	13 (76.5%)
Maybe	4 (23.5%)
No	0 (0.0%)

Clinical and functional outcomes improved significantly at 6 months. Mean leg pain VAS decreased from 8.47 ± 1.91 preoperatively to 2.24 ± 2.02 (p < 0.001), and low back pain from 7.82 ± 2.30 to 2.06 ± 1.25 (p < 0.001). The ODI improved from 53.53 ± 19.06 to 29.18 ± 16.25 (p < 0.001). SF-12 PCS increased from 30.34 ± 7.18 to 38.22 ± 7.91 (p = 0.018), and MCS from 36.68 ± 9.49 to 43.38 ± 13.78 (p = 0.046). These findings are summarized in Table 3.

**Table 3:** Clinical and functional outcomes (mean ± standard deviation).

Variable	Pre-operative	Post-operative (6 months)	p-value
Leg pain (VAS)	8.47 ± 1.91	2.24 ± 2.02	**<0.001**
Low back pain (VAS)	7.82 ± 2.30	2.06 ± 1.25	**<0.001**
ODI (Total score)	53.53 ± 19.06	29.18 ± 16.25	**<0.001**
PCS (SF-12)	30.34 ± 7.18	38.22 ± 7.91	**0.018**
MCS (SF-12)	36.68 ± 9.49	43.38 ± 13.78	**0.046**

## 5 Discussion

This single-center series suggests that full-endoscopic trans-pars interarticularis extraforaminal discectomy provides effective decompression with favorable early clinical outcomes in selected patients with foraminal or extraforaminal lumbar disc herniation (LDH). At 6 months, we observed large and statistically significant reductions in leg and back pain, substantial improvement in disability and health-related quality of life, and high patient satisfaction, in the absence of intraoperative complications or early recurrent herniation.

Our findings complement and extend the initial published study on this technique (Greil et al., 2023), which described a full-endoscopic trans-pars interarticularis approach in 14 patients with far-lateral LDH, achieving mean improvements of 4.3 points in leg pain VAS, 2.6 points in back pain VAS, and 20.6 points in ODI at a mean follow-up of 21.9 months, with no major complications. In our cohort, mean leg pain VAS improved by 6.23 points (from 8.47 ± 1.91 to 2.24 ± 2.02) and low back pain VAS by 5.76 points (from 7.82 ± 2.30 to 2.06 ± 1.25) at 6 months (p < 0.001 for both), with ODI decreasing by 24.35 points (from 53.53 ± 19.06 to 29.18 ± 16.25; p < 0.001). Even though the sample size is limited (17 patients), our study included multiple lumbar levels, both foraminal and extraforaminal herniations, and systematically assessed satisfaction and quality-of-life metrics. Our series thus supports the feasibility and safety of this trans-pars approach and suggests that the magnitude of pain and disability improvement is at least comparable, and numerically somewhat greater, than that reported in the original description.

The outcomes observed are also in line with other endoscopic techniques targeting lateral lumbar pathology. A previous study reported that percutaneous endoscopic discectomy using an “extraforaminal targeted fragmentectomy” technique in 41 patients with extraforaminal LDH reduced radicular VAS from 8.6 to 1.9 and ODI from 66.3 to 11.5, with 92% overall satisfactory outcomes (Choi et al., 2007). A study compared endoscopic and microscopic extraforaminal discectomy in 118 patients and found similar improvements in VAS and ODI and comparable complication rates, while the endoscopic technique offered the advantages of a minimally invasive approach (Aydın et al., 2020). More recently, a study demonstrated that an extraforaminal full-endoscopic approach with foraminoplasty yields significant pain relief and functional gains with few complications in patients with lateral lumbar herniation or stenosis (Bergamaschi et al., 2023). Our VAS and ODI changes fall within the range reported in these series, supporting the concept that our technique can provide robust symptom relief while limiting tissue disruption.

There is now solid evidence that transforaminal full-endoscopic discectomy is an established alternative to standard microdiscectomy. In a systematic review and meta-analysis of 1527 patients, transforaminal endoscopic discectomy was found to provide leg pain and ODI improvements, complication rates, and recurrence rates comparable to conventional microdiscectomy, while also shortening hospital stay (Zhang et al., 2018). However, in routine practice the classic Kambin's triangle route brings the working cannula very close to the exiting nerve root and dorsal root ganglion, which form the hypotenuse of the triangle, and anatomical studies have shown that this working corridor may become particularly narrow at lower lumbar levels and in degenerative foraminal stenosis, thereby reducing the safety margin for instruments and endoscopic manipulation (Ozer et al., 2017; Sakane, 2017; Uchikado et al., 2020). Clinically, this limited space is reflected by dysesthesia from dorsal root ganglion irritation, one of the most frequent benign adverse events after transforaminal endoscopy, reported in roughly one-fifth of cases overall and especially in patients with foraminal stenosis or recurrent herniation, where the cannula must be advanced directly alongside the exiting root (Lewandrowski et al., 2020a). Thus, the trans-pars interarticularis extraforaminal trajectory can be considered an alternative of the transforaminal technique that preserves the minimally invasive advantages of endoscopic surgery while intentionally shifting the bony entry point away from the dorsal root ganglion and providing more direct access to the extraforaminal compartment (Musharbash and Lee, 2023). Notably in our study, postoperative leg dysesthesia occurred in 11.8% of patients but was not associated with new motor deficit or the need for reoperation, with all cases being transient and resolving spontaneously. This suggests that, while the trans-pars approach does not completely eliminate DRG irritation, it permits safe neural decompression with an acceptable profile of largely transient sensory complaints. Recent modifications such as intertransverse or extraforaminal “outside-in” endoscopic approaches have similarly sought to increase the distance between the working sheath and the exiting root to reduce dysesthesia, underscoring that optimization of the lateral access trajectory remains an active area of innovation (Musharbash and Lee, 2023; Lee et al., 2024; Chang, 2025).

The substantial improvements in disability and quality of life observed in our series are clinically meaningful. The Oswestry Disability Index (ODI) is a widely used, validated measure with good construct validity, internal consistency, and responsiveness in patients with low back pain and lumbar spinal disorders, including populations undergoing lumbar surgery (Vianin, 2008; Koivunen et al., 2024). In a large series of patients treated with transforaminal endoscopic decompression for lateral recess and foraminal stenosis, it estimated minimal clinically important differences (MCID) of 2.5-3.5 points for VAS and 15-16.5 points for ODI (Lewandrowski et al., 2020b). The mean reductions in our cohort (approximately 6.2 points for leg pain and 24.4 points in ODI) exceed these MCID thresholds, indicating that the majority of patients experienced improvements that are not only statistically significant, but also perceptible and relevant in daily life.

An important technical principle of our technique is that the osseous work is intentionally minimal and focal, rather than resecting the pars or facet in a subtractive fashion, we perform a small cortical window at the pars-transverse process junction to gain access. The pars continuity and the facet complex are preserved, and the extent of bony removal is limited to what is required for endoscopic visualization and safe fragment extraction. While any pars work raises a theoretical concern for iatrogenic weakening, available clinical data suggest that postoperative stability is related to the amount of isthmus or pars removed (Sari et al., 2020), supporting the importance of our minimal invasive approach. Accordingly, we view this trans-pars corridor as a pars-preserving approach; however, prospective studies with longer follow-up, including quantification of bony work and radiographic surveillance for stability, are warranted.

This study has several limitations. Its small sample size limit the precision of estimates and preclude multivariable analysis of predictors of outcome or dysesthesia. There is no control group treated with microdiscectomy, standard transforaminal endoscopy, or other minimally invasive techniques, so direct comparisons of efficacy, complication rates, and resource utilization cannot be made. The follow-up period of 6 months is relatively short and may not capture delayed events, including spondylolysis or late segmental instability. Finally, this is a single-center and single-surgeon experience, which may limit the generalizability of our results to other settings and surgeons.

Despite these limitations, our findings add clinical outcome data to the growing body of work on the trans-pars endoscopic approach and support its role as a safe and effective option for the treatment of foraminal and extraforaminal LDH, particularly when conventional transforaminal technique is constrained or considered high risk. Future prospective, comparative studies with larger cohorts, standardized radiological assessments, and longer follow-up are warranted to better define the relative advantages of the trans-pars approach within the spectrum of modern endoscopic strategies for foraminal or extraforaminal LDH.

## 6 Conclusion

Endoscopic trans-pars discectomy appears to be a safe and effective option for foraminal and extraforaminal lumbar disc herniation, providing substantial reductions in leg and back pain, disability, and quality-of-life impairment, with high patient satisfaction and no major complications or early recurrences at 6 months. Larger prospective, comparative studies are needed to confirm these findings and define the optimal indications for this trans-pars endoscopic approach.

## Author contributions

Conceptualization: YJ, MB. Data curation: YJ, GS, MB, AHH. Formal analysis: YJ, AHH. Investigation: YJ, MB, AHH. Writing original draft: YJ. Writing-review and editing: YJ, MB, GS, AHH, RaM, RMou, KA. Supervision: YJ, MB. Final approval of the manuscript: all authors.

## Funding

This research did not receive any funding.

## Declaration of competing interest

The authors declare that they have no known competing financial interests or personal relationships that could have appeared to influence the work reported in this paper.
